# A Prospective Study Comparing the Infection Rate Between Buried vs Exposed Kirschner Wires in Hand and Wrist Fixations

**DOI:** 10.7759/cureus.36558

**Published:** 2023-03-22

**Authors:** Shalimar Abdullah, Elaine Zi Fan Soh, Choong Jin Ngiam, Jamari Sapuan

**Affiliations:** 1 Hand and Microsurgery Unit, Department of Orthopaedics and Traumatology, National University of Malaysia, Kuala Lumpur, MYS

**Keywords:** exposed k-wires, buried k-wires, percutaneous pinning, infection, osteomyelitis

## Abstract

Introduction: Kirschner wires (K-wires) are used in fracture fixations but are often associated with pin tract infections. This prospective study compared the infection rate between buried and exposed K-wires in closed injuries of the wrist and hands in individuals with no comorbidities.

Methods: Fifteen patients were recruited with a total of 41 K-wires (21 buried K-wires; 20 exposed K-wires). Clinical and radiographic evidence of infection was assessed at three months based on the Modified Oppenheim classification.

Results: Two out of 21 wires in the buried group developed grade 4 infection, while 20 wires in the exposed group did not have any significant infection. No significant difference in infection rate based on K-wire size or number in both groups.

Conclusion: There is no significant difference in infection rate between buried and exposed K-wires in healthy individuals with closed injuries of the wrist and hand.

## Introduction

Kirshner wires or commonly known as K-wires were first introduced by Martin Kirschner in 1909 and since then have been widely used in various orthopedic surgeries [1,2]. After many years of advancement and perfecting the use of this device, K-wires became one of the commonest implants utilized in hand fixations since 1937 [2]. Despite the smooth design of these wires, they provided adequate bone stabilization in fracture fixations and less soft tissue trauma during insertion [2,3]. The cost-effective treatment with K-wires allows the versatile use of this implant and makes it available in most Orthopedics treatment centers as compared to other more costly choices of implants. In comparison, plating and screws are more expensive compared to K-wires which makes them less affordable and less accessible to patients that are unable to afford such implants [4]. K-wire sizes for hand and wrist fixations may vary and range from 1.0 to 1.8mm depending on bone morphology and fracture configuration.

After K-wire placements in fracture fixations, they may be left buried under the skin or left exposed outside the skin. Exposed K-wires commonly necessitate frequent dressing over exposed wire sites as they pose a potential risk of pin tract infection. Both techniques of K-wires placement are usually left in situ for a duration of 3-6 weeks depending on the healing process and are then removed prior to the initiation of physiotherapy [3]. K-wires are usually removed in the daycare clinic or operating theatre and may occasionally require early removal due to complications.

The commonest complication of K-wire fixations is notably pin site infection which has been reported in several studies to be as high as 4-20% [3,5-7]. A possible contributing factor to the incidence of infection could be due to thermal and mechanical injury to soft tissue from the twisting of wires during insertion [8]. Furthermore, severe infections such as abscesses and osteomyelitis have also been reported [5,6]. Early removal may result in fracture destabilization. Other known complications of K-wire fixations are tendon rupture, septic arthritis, nerve injury, K-wire migration, and loosening [9]. K-wire site infection is commonly treated with antibiotics and early pin removal and occasionally may necessitate surgical debridement.

To the best of our knowledge, to date, there are minimal studies done in Asia that compare the outcome and infection rate between buried and exposed K-wires in hand and wrist fixations, nor are there any prospective study in Asia that compares the outcome and infection rate between the two techniques. According to research done outside of Asia, there seem to be conflicting results when comparing infection rates between these two methods of K-wire fixations in the hand and wrist, thus no general consensus if K-wires should be left buried or exposed [10]. A prospective study done in the UK showed a statistically significant risk of infection in exposed K-wires as compared to buried wires [11]. Similarly, a few retrospective studies pointed out similar results in exposed K-wires [3,12]. Other retrospective studies found that there was no significant difference between the two groups [1,7,13,14]. The limitation of most retrospective studies published previously does not provide concrete evidence to support the difference between the two groups' possibility of perpetual bias as the patients were not assessed clinically and data records may not be accurate. Closed and open injuries of the hand and wrist were included in previously mentioned studies.

Therefore, the objective of our study was to look at the overall difference in presence of infection between buried and exposed K-wires, to look at the risk of infection based on K-wire size and anatomical bony location of fixation exclusively in closed injuries of the wrist and hand in healthy individuals.

## Materials and methods

Patients with closed bony and/or ligamentous injury of the hand and/or wrist in our center were identified. This study was approved by the institution's ethical committee. Patients that enrolled were aged 18 and above, healthy with no comorbidities, with closed injuries involving distal end radius, distal end ulna, carpal, metacarpal, phalanx, and ligament injury of the hand and wrist requiring K-wire fixation. Patients that were excluded were aged below 18, open ligamentous or bony injuries of the wrist and hand that necessitate a K-wire fixation, a local operative site with dermatological infection, diabetics or immunocompromised patients, fixation(s) that required more than four K-wires in an ipsilateral wrist or hand and those followed up at a different hospital post-operatively. Those eligible for elective K-wire fixation were randomized using an online generated program into two groups after consent was obtained. The intervention group consists of patients planned for buried K-wires and the control group consists of patients planned for exposed K-wires. No post-randomization blinding was done for either the patient, care physician, or investigator physician.

Surgery was carried out in a laminar flow operation theatre by either an orthopedic registrar or a consultant. All patients were given a single dose of IV cefuroxime 1.5g half an hour before surgery. The surgical field was cleaned and draped in a sterile manner. Post-operatively, K-wire size, number, and anatomical location of K-wire placement was documented. Exposed wires were bent at right angles close to the skin edge to avoid migration and to provide a tension-free interface between the skin and K-wire. Exposed ends were capped with a branula stopper to prevent sharp injury. The surrounding exposed wire was covered with diluted povidone-soaked ribbon gauze (povidone-iodine solution B.P. 10% w/v diluted in a ratio of 1:1 with sodium chloride 0.9%). Patients with exposed wires were instructed to adhere to strict two days once pin site dressing at the nearest clinic. Wires that were buried were cut deep to ensure that the wires were placed beneath the subcutaneous plane. No stitches were done. Both groups of patients were followed up at our hand clinic for three months duration. All patients were reviewed in the third and sixth weeks to assess both clinical and radiographic evidence of pin site infection based on the Modified Oppenheim classification (Table 1) [11]. Infection was deemed present from grade 2 onwards [11]. K-wire removal was carried out by the sixth to eighth week post-operatively.

**Table 1:** Modified Oppenheim classification

Grades	Clinical findings	Treatment
1	Slight discharge and redness around pin	Local pin and wound care
2	Redness and tenderness in soft tissues with or without discharge of pus	Local pin and wound care + oral antibiotics
3	As for grade 2 but with failure to improve with local care and antibiotics	Infected pins removed + oral antibiotics
4	Severe soft tissue involvement affecting more than one pin	Infected pins removed + oral antibiotics
5	As for grade 4 but also with bone involvement visible on X-ray	Pins removed + curettage of bone
6	A sequestrum has formed within the bone and a persistent sinus has developed	Further surgery required to eradicate problem

## Results

**Figure 1:**
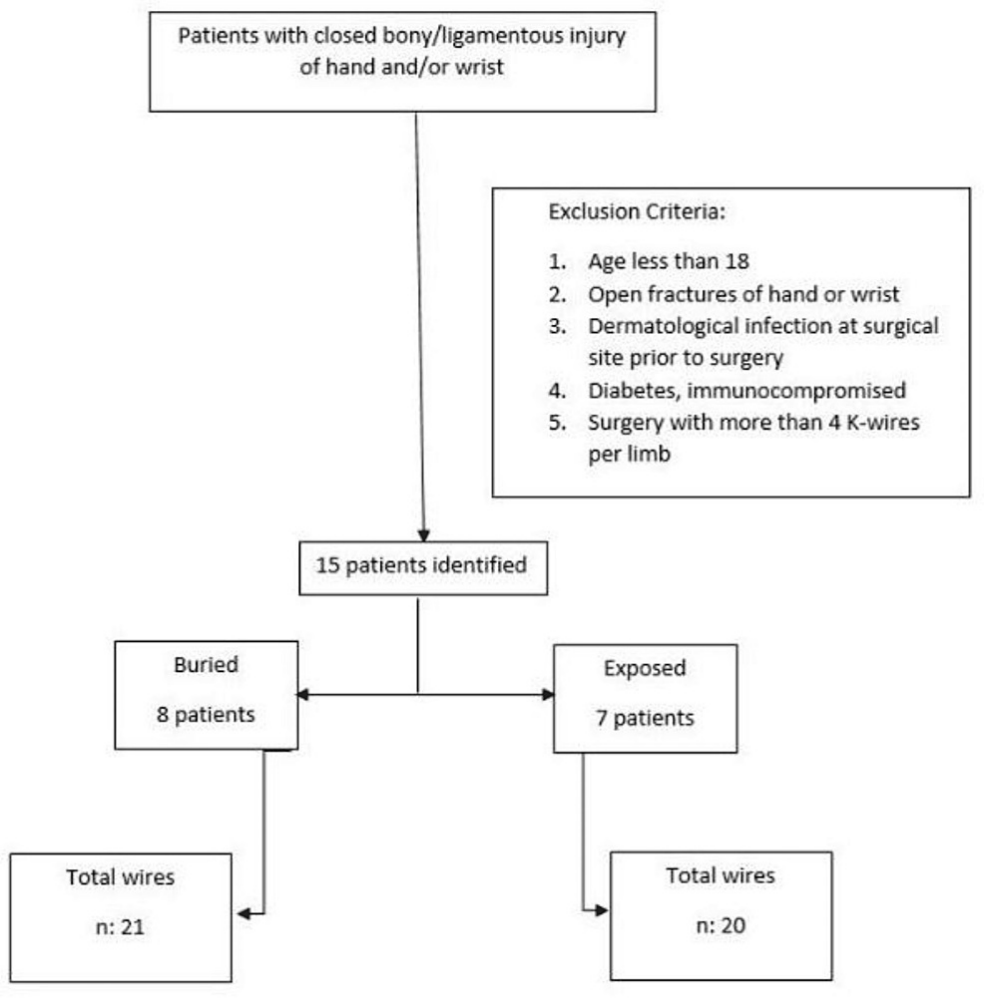
Flow chart showing study population distribution

The final study population involved 15 patients of which eight patients were treated with a total of 21 buried K-wires and seven patients were treated with a total of 20 exposed K-wires (Figure 1). The total number of wires demonstrated in our study population was 41.

The demography of our patients is depicted in (Table 2). The mean number of wires in the buried group consist of 2.62 while the exposed group was 2.85. The mean age in the buried group was 36.3 years while the exposed group was 36.7 years. Gender distribution in the study group consisted of 14 male patients and one female patient.

**Table 2:** Demographic data of patients in both groups

	Total	Buried	Exposed
No. of Patients	15	8	7
Mean Age (range)	36.55 (18-61)	36.3 (18-61)	36.7 (23-50)
Gender: M/F	14/1	7/1	7/0

The number of cases based on wiring method and anatomical location is as per Table 3.

**Table 3:** Number of cases based on wire location between the two wiring methods

Methods of wiring	Wire location				
	Carpal	Distal radius/ulna	Metacarpal	Phalanx	Total cases
Buried	3	3	2	1	9
Exposed	1	3	4	3	11
Total	4	6	6	4	20

A range of wire sizes was used from 1.0mm to 2.0 mm with 1.2 mm and 1.8mm being the most commonly used wires (Table 4).

**Table 4:** Number of wires based on wire size between the two wiring methods

Methods of wiring	Wire size (mm)						
	1	1.2	1.4	1.6	1.8	2.0	Total wires
Buried	2	4	6	5	4	0	21
Exposed	4	6	5	0	4	1	20
Total	6	10	11	5	8	1	41

As for the grade of infection (Table 5), there were two wires that were from a closed carpometacarpal (CMC) dislocation that developed a grade 4 infection. The two infected wires were in close proximity to each other and were from the same patient. The patient required surgical debridement, removal of wire, and intravenous antibiotics. Save and except for the abovementioned grade 4 infection patient, other patients from the buried group did not develop signs of infection. Patients in the exposed group did not develop any serious infection despite showing grade 1 infection which resolved after local pin and wound care.

**Table 5:** Number of wires that were infected based on Modified Oppenheim Grade between the two wiring methods

Methods of wiring	Grade of infection							
	Non-infected		Infected					
	None	Grade 1	Grade 2	Grade 3	Grade 4	Grade 5	Grade 6	Total wires
Buried	19	0	0	0	2	0	0	21
Exposed	0	20	0	0	0	0	0	20
Total	19	20	0	0	2	0	0	41

The average number of weeks before buried K-wires were removed in the daycare operation theatre was 6.6 weeks, as opposed to 6.4 for non-buried K-wires. The overall average of wire removal was at about the sixth week (Table 6).

**Table 6:** Number of patients in which wires were removed on a specific week between the two wiring methods

Methods of wiring	Weeks of removal				
	5^th^	6^th^	7^th^	8^th^	Total patients
Buried	1	3	2	2	8
Exposed	0	5	1	1	7
Total	1	8	3	3	15

Overall, there was no clinically significant difference in the rate of infection between the patients in the buried and exposed K-wire group.

## Discussion

The outcome of buried K-wires versus exposed K-wires in hand and wrist fixation still remains a debatable topic. The decision of either technique is dependent on the surgeon’s preference as both techniques have their advantages and disadvantages. Exposed wires carry a higher possible risk of pin-related infection, however, are easily removed in the clinic setting. Buried wires require additional cost and utilization of daycare operating theatre for removal. To the best of our knowledge, there have been conflicting results when comparing the infection rates between buried and exposed K-wires. According to Hargreaves et al., the study showed that 34.5% significant risk of infection in exposed K-wires as compared to a 7% risk of infection in buried K-wires [11]. Similarly, a few retrospective studies have shown similar outcomes in exposed K-wires [3,12]. Other retrospective studies found there were no significant differences between the two groups [1,7,13,14].

Our study did not demonstrate a significant risk of infection in the exposed K-wires group. The hand and wrist are constantly exposed to our surrounding and pose a risk of developing local pin site infection, hence patients in the exposed K-wire groups were advised to adhere to strict local dressing protocol post-operatively which consist of once every two days dressing during the six weeks of observation. The importance of dressing compliance may have prevented the risk of infection in the control group, however, we cannot conclude that our study can justify the comparison of both groups as our study is limited by a small sample size and short duration of observation. There is no study to define the frequency of dressing required for hand and wrist K-wires. A prospective study on external fixator pins has shown no difference between daily and weekly dressing [15]. Bending exposed ends of K-wires close to the skin can ease skin tension and prevent wire migration. The lesser the skin tension, the lesser soft tissue irritation at the wire skin interface. The importance of bending wire at ends and placement of endcap prevents the risk of injuring oneself and accidental removal of wire when unknowingly hooked onto one’s clothing. A cost-effective technique demonstrated by Ibrahim with the use of rubber stoppers from IV drip bottles prevents wire motion and holds dressing in place showed a lesser risk of K-wire-related infection [16]. Exposed ends of the wire that are constantly in contact with external surrounding caters to the potential pathway of infection into deeper tissue. The use of povidone dressing to envelope exposed ends of wires in our control group would have prevented potential source of infection similarly as demonstrated in a pilot study by Grant [17]. Dressing protocols and appropriate solution for pin site dressing is still debatable as there has been no study to conclude which protocol or solution is superior due to many confounding factors [18-20].

Previous studies attempted to find a correlation between medical co-morbidities and infection risk however failed to yield any significance among the two groups [3,5,21]. By further eliminating potential biases of medical co-morbidities and local skin infection that may increase the chance of developing infection, we found no correlation of risk of K-wire infection between the two groups as our study population included were healthy individuals with no underlying medical illness or had a local skin infection, to begin with. We can safely say that options between buried or exposed K-wires for closed injuries in the hand and wrist in a healthy individual can be offered as either technique does not significantly increase the risk of infection.

The duration of K-wire left in situ for fixation in our study averaged a mean of six weeks and also did not yield a significant risk of infection between the two groups. One patient with two buried K-wires for closed dislocation of the left fifth CMC joint had developed a grade 4 infection which was complicated by a hypothenar abscess in the eighth week. Two other patients, in which one had two buried wires and the other had two exposed wires placed over the carpal bones similarly had their wires removed at the eighth week but did not demonstrate any signs of significant local infection. Botte found that infection developed at an average of 10 weeks while Hargreaves demonstrated that despite the removal of K-wires by the sixth week still pose the risk of developing pin-related infection even though there is insufficient time to develop biofilm along exposed ends of wires [11,22,23]. This is further supported by Padmanabhan when he demonstrated a lesser infection rate when K-wires for distal end radius fractures were removed earlier by the fourth week [24]. However, the results from the aforementioned studies did not stratify patients based on closed or open injuries. Our study population was confined to closed injuries to the wrist and hand which may plausibly yield discrepancy in the results obtained.

Subgroup analysis of K-wire sizing and infection risk between the two groups did not demonstrate any significance. We were unable to identify any correlation as the numbers of K-wires sizes were not equally distributed and overall there was no significant infection risk between the two. A larger sample size would be required to demonstrate this correlation.

More wires used for fixation would potentially increase the risk of pin-related infection. We limit our inclusion criteria to a maximum of four wires in both groups, however, did not demonstrate any significant risk of infection. The only infected case in the buried group had two wires placed in one location but inadvertently developed an infection. We could not find any correlation between the number of wires and the risk of infection in both groups possibly all cases included were closed injuries in healthy individuals. A retrospective study similarly found no correlation between the number of wires and infection, despite including cases with open injuries [5].

In subgroup analysis comparing the anatomical location of the wire, we identified only one patient with severe infection following buried K-wiring of the left fifth metacarpal base. The patient was treated surgically and given a week’s course of IV antibiotics. Several retrospective studies identified an increased incidence of infection in metacarpal K-wiring [3,5,7]. Patients with buried K-wires have the liberty of performing finger exercises as the movement of metacarpal wires is masked by thick soft tissue surrounding the embedded wires. Micromotion between the wire and surrounding soft tissue interface may have resulted in excessive soft injury leading to deep-seated infection in buried metacarpal wires [3]. Periarticular placement of wires may also be associated with a higher risk of infection due to increased soft tissue motion around joints [3]. We could not identify other plausible factors contributing to his infection apart from that the surgery required open reduction before K-wire placement which could possibly relate to difficult reduction and prior multiple drilling attempts.

The strength of our study includes the prospective nature of the study and assessment by a single observer. Strict selection criteria for enrolment such as limiting to four (4) K-wires, healthy individuals with no underlying co-morbidities, and closed injuries allow us to analyze and compare homogeneity characteristics. Limitations of our study include the short duration of the study with a small sample size due to homogeneity sampling. However, this is a general limitation of studies with narrow inclusion criteria. The small sample size is attributed due to the majority of hand and wrist injuries being treated with screws and plates at our center which provides Hand and Microsurgery expertise as compared to other district hospitals. Overall, our study shows no significant risk of infection between the two groups exclusively in healthy individuals. The study can be used as a guide to further replicate similar studies in the district hospitals as K-wiring of hand and wrist is still a common practice.

## Conclusions

This study had narrow inclusion criteria, thus minimizing confounders, and making interpretation clearer. The results showed that regardless of the position of wires and methods of fixation, the outcome was good if patients were generally healthy and had minimal co-morbid. This is a clinically useful finding. There is no clinical significance of infection rate between buried and exposed K-wires in healthy individuals with closed injuries of the wrist and hand. The infected K-wires in the buried group were localized at the metacarpal thus we recommend caution and closer observation when leaving K-wires in situ in metacarpals.
